# The Ocular Benefits of Estrogen Replacement Therapy: A Population-Based Study in Postmenopausal Korean Women

**DOI:** 10.1371/journal.pone.0106473

**Published:** 2014-09-11

**Authors:** Kyung-Sun Na, Dong Hyun Jee, Kyungdo Han, Yong-Gyu Park, Man Soo Kim, Eun Chul Kim

**Affiliations:** 1 Department of Ophthalmology, College of Medicine, The Catholic University of Korea, Seoul, Korea; 2 Department of Biostatistics, The Catholic University of Korea, Seoul, Korea; Save Sight Institute, Australia

## Abstract

**Purpose:**

To elucidate the prevalence of cataract, glaucoma, pterygia, and diabetic retinopathy among Korean postmenopausal women with or without estrogen replacement therapy (ERT).

**Methods:**

A cross-sectional, nationally representative sample from the 4th Korea National Health and Nutrition Examination Survey (KNHANES IV) (2007–2009) was used. Participants were interviewed for the determination of socioeconomic and gynecologic factors. Each woman also underwent an ophthalmologic examination and provided a blood sample for risk factor assessment.

**Results:**

Of 3968 postmenopausal women enrolled, 3390 had never received estrogen, and 578 were undergoing estrogen treatment. After adjusting for age, diabetes, hypertension, high cholesterol levels, and high low-density lipoprotein levels, the prevalence of anterior polar cataract, retinal nerve fiber layer (RNFL) defect, and flesh pterygium was higher in the non-ERT group (OR, 3.24; 95% CI, 1.12–9.35, OR 1.70; 95% CI, 1.04–2.78, OR 3.725; 95% CI, 1.21–11.45, respectively). Further, the prevalence of atrophic pterygium was lower in the non-ERT group compared to that in the ERT group (OR, 0.21, 95% CI, 0.07–0.63).

**Conclusions:**

These data suggest that ERT has a protective effect against the development of anterior polar cataract, flesh pterygium, and RNFL defect.

## Introduction

Estrogen replacement therapy (ERT) is widely used for controlling menopausal symptoms and has also been used for the management and prevention of cardiovascular disease [1]–[3], osteoporosis [1], [4], and dementia [1], [5], [6]. Estrogen affects vascular tone and blood flow in organs and tissues [7] and also exerts neuroprotective effects via nonvascular mechanisms [8].

Significant gender differences in the incidence of various ocular diseases raise the possibility that estrogens may have effects on the eye. Estrogen receptor mRNA was shown to be expressed in the retina, retinal pigment epithelium (RPE), ciliary body, iris, and lens epithelium of human cadavers, suggesting that estrogen may also play a role in the pathogenesis of ocular diseases in humans [9], [10]. The serum level of sex hormones has been correlated with dry eye disease [11], and a single nucleotide polymorphism (SNP) in estrogen was found to be associated with glaucoma [12]. Therefore, we postulated that ERT affects the prevalence of ocular conditions in postmenopausal women. Previous studies assessed the effects of postmenopausal ERT on lens opacities [13], [14], intraocular pressure [15]–[17], and maculopathy [18]. Two large cross-sectional, population-based studies found associations between the use of hormone replacement therapy and cataract [13], [14]. Most of these studies have the limitation of selection bias because they used data from a single hospital with a relatively small number of subjects or they were population-based studies conducted only in Western countries. Few studies have investigated the prevalence of general ocular diseases among postmenopausal women with or without ERT by using Asian population-based data.

This cross-sectional study was based on data collected during the period from 2007 to 2010 as part of the Korean National Health and Nutrition Examination Survey (KNHANES) conducted by the Division of Chronic Disease Surveillance under the guidance of the Korea Centers for Disease Control and Prevention. We analyzed these data to assess the relationship between exogenous estrogen exposure and the prevalence of ocular diseases (cataracts, glaucoma, pterygia, and diabetic retinopathy) among postmenopausal Korean women.

## Methods

### Study Subjects

The KNHANES survey was composed of 3 parts: a health interview survey, a health examination survey, and a nutrition survey. This concerted effort was a nationwide representative study of non-institutionalized civilians, based on a stratified, multistage, probability-sampling design with a rolling survey sampling model. The sampling units were defined on the basis of household unit data obtained from the 2005 National Census Registry, including those for geographic area, sex, and age.

The total population included in this survey for primary sampling was 15712. The subjects were randomly sampled throughout South Korea and were included in the Health Interview Survey. From among this total sample population, we selected 9966 adult women who had completed the Health Examination Survey and underwent ophthalmologic examinations. Subjects who were not experiencing menopause, who had been diagnosed with any systemic inflammatory or infectious disease other than diabetes, and who had not undergone ovariectomy after a premenopausal hysterectomy were excluded (n = 5739); 259 subjects were excluded because of incomplete medical data. For those women who were not officially classified as post-menopausal, an age ≥65 years was considered sufficient proof of a postmenopausal state. Ultimately, 3968 postmenopausal women were included in this study. The ERT group included women who received only estrogen oral therapy for at least 12 months prior to enrolment in the study. In order to assess the relationship between ocular conditions and estrogen per se, women taking estrogen/progesterone combination therapy were excluded. This survey was reviewed and approved by the Institutional Review Board of the Korea Centers for Disease Control and Prevention.All participants provided written informed consent.

### Procedures

Questionnaires were used to collect a detailed medical history, which included the age at menarche and menopause, the reason for menopause, and any history of hysterectomy and/or ovariectomy, pregnancy, parity (the number of live-born children), oral contraceptive use, ERT use, as well as the duration of any ERT treatment. Surgical menopause was defined as the cessation of menstrual periods after an ovariectomy. Women who stated that they had entered menopause naturally, as well as those who had undergone hysterectomy without ovariectomy, were considered postmenopausal. The general medical history and medication information were also recorded.

All anthropometric measurements were obtained by a specially trained examiner. Waist circumference was measured in a horizontal plane at the level of the midpoint between the iliac crest and the costal margin. Body mass index (BMI) was calculated as the individual's weight in kilograms divided by the square of the individual's height in meters. Systemic hypertension was defined as measured systolic blood pressure >160 mmHg and/or diastolic blood pressure >90 mmHg or if the patients were currently using systemic antihypertensive drugs. Blood pressure was measured in a seated position and after at least 5 min of rest. Diabetes was defined as a fasting blood sugar level > 126 mg/dL or if the patient was currently using antidiabetic medication. Peripheral blood was obtained after fasting for a minimum of 8 h.

### Ophthalmologic examinations

Visual acuity was tested by using a LogMAR scale chart (Jin's vision chart, Seoul, Korea); refractive error was estimated by using an autorefractor-keratometer (KR8800; Topcon, Tokyo, Japan). The anterior segment (e.g., pterygia and cataract) and intraocular pressure (IOP) were measured upon completion of the slit-lamp examination (Haag-Streit model BQ-900; Haag-Streit AG, Koeniz, Switzerland). A pterygium was defined as a radially oriented fibrovascular lesion crossing over the nasal or temporal limbus. Grading was based on the visibility of the underlying episcleral blood vessels [19]. An atrophic pterygium was defined as a pterygium that allowed the clear discernment of the underlying episcleral vessels. A flesh pterygium was defined as a thick pterygium that did not allow the visualization of the episcleral vessels.

The subtype of cataract present (i.e., nuclear, cortical, posterior subcapsular, anterior polar, or mixed) was noted. Subjects who were aphakic, pseudophakic, and those with different cataract types in either eye were excluded from the analysis to prevent bias.

Digital fundus images were taken by using a non-mydriatic fundus camera (TRC-NW6S, Topcon) and a Nikon D-80 digital camera (Nikon, Tokyo, Japan). The horizontal and vertical cup-to-disc (C/D) ratios were determined based on the fundus photographs. For each participant who had a history of diabetes mellitus or a random blood glucose level of ≥200 mg/dL and those suspected to have diabetic retinopathy based on the non-mydriatic fundus photographs, 7 standard photographs were obtained from each eye after pharmacological pupil dilation, as recommended by the Early Treatment for Diabetic Retinopathy Study. Diabetic retinopathy was defined as the presence of 1 or more retinal microaneurysms or retinal blot hemorrhages with or without more severe lesions (hard exudates, soft exudates, intraretinal microvascular abnormalities, venous bleeding, new retinal vessels, or fibroglial proliferation).

### Statistical Analysis

The data are expressed as numbers and percentages (categorical) or the mean ± standard deviation (continuous). The demographic and biochemical characteristics of the study population and the prevalence of ophthalmic findings according to ERT status were compared by using the independent 2-sample t test for continuous variables and the χ^2^ test for categorical variables. Multivariate adjusted logistic regression analysis was conducted to examine the odds ratio (OR) and 95% confidence interval (CI) for the association of ERT with cataract, glaucoma and pterygium. Patient age and the presence of diabetes, hypertension, high cholesterol levels, or high LDL levels were adjusted to compare the effect of ERT after eliminating the effect of well-known or possible risk factors in the pathogenesis of various ocular diseases. Further, all of the factors that were identified as general characteristics that were affected by ERT in the univariate analysis were included in the multivariate analysis.

The prevalence of cataract, and glaucoma, were estimated for the overall study population. The presence of diabetic retinopathy was analyzed only for those with diabetes. The percentages of atrophic and flesh pterygia were determined in the population with pterygium.

Notably, the KNHANES included weights to compensate for the complex sampling design and to represent the Korean population accurately. In this study, all analyses were performed by using version 9.2 SAS software (SAS Institute, Cary, NC, USA) to account for the complex sampling design and to provide nationally representative prevalence estimates. A p value <0.05 was considered statistically significant.

## Results

### Baseline Characteristics

Among the 3968 postmenopausal women enrolled, 3390 had never received estrogen, and 578 were taking estrogen at the time of the study. All of the women in the ERT group took oral estrogen therapy. The demographic and biochemical characteristics are described in Table 1.

**Table 1:** Baseline characteristics of the study participants.

	ERT (n = 578)	Non-ERT (n = 3390)	P-value
Age	58.3±0.3	63.9±0.3	<.0001
Body mass index	24.0±0.1	24.2±0.1	0.3211
Waist circumference	81.0±0.4	82.5±0.2	0.0021
Cholesterol	198.5±1.8	202.2±0.9	0.0551
High-density lipoprotein	55.0±0.7	52.1±0.3	<.0001
Triglyceride	122.2±3.5	141.9±2	<.0001
Low-density lipoprotein	124.2±1.5	127.5±0.8	<.0001
Hemoglobin	13.3±0.02	13.1±0.04	<.0001
Hematocrit	39.9±0.1	39.3±0.1	<.0001
Blood urea nitrogen	15.0±0.2	15.5±0.1	0.0193
Creatinine	0.714±0.005	0.728±0.004	0.0253
Vitamin D	19.5±0.4	18.7±0.2	0.0581
Hypertension	41.1(2.5)	52.7(1.1)	<.0001
Osteoporosis	16.2(1.8)	39.0(1.1)	<.0001

The values represent the means ± SD or percentages (SE).

The independent 2-sample t test was used for continuous variables and the χ^2^ test was used for categorical variables.

ERT, estrogen replacement therapy.

### Associated Ocular Disorders

Prevalence (Table 2) and logistic regression models (Table 3) were used to evaluate the associations between various baseline factors and the presence of cataract, glaucoma, pterygia, and diabetic retinopathy.

**Table 2:** Ophthalmic findings in postmenopausal Korean women with or without ERT.

	ERT (n = 578)	Non-ERT (n = 3390)	P-value
**Cataract**§	44.2(2.6)	63.3(1.6)	<.0001
Cortical	10.1(1.5)	11(0.9)	0.5654
Nuclear	26.4(2.3)	31.6(1.5)	0.0276
Anterior polar	0.4(0.2)	1.3(0.2)	0.0186
Posterior subcapsular	0.6(0.4)	0.9(0.2)	0.6174
Mixed	4.2(1.1)	11.1(0.8)	0.0001
**Glaucoma**			
Intraocular pressure*	14.8±0.1	14.4±0.1	0.004
Increased intraocular pressure*†	0.7(0.4)	0.4(0.1)	0.3271
Cup-to-disc ratio*			
*vertical*	0.378±0.007	0.391±0.004	0.0769
*horizontal*	0.382+0.007	0.392±0.003	0.1793
Abnormal cup-to-disc ratio*‡	14.4(1.7)	17.2(0.8)	0.1487
Disc hemorrhage	0.6(0.3)	0.7(0.2)	0.9211
Retinal nerve fiber layer defect	3.2(0.8)	5.5(0.5)	0.0243
**Pterygium**	4.5(1.1)	11.9(0.7)	<.0001
Atrophic	87.9(6.1)	50.1(3.3)	<.0001
Flesh≠	12.1(6.1)	41.3(3.1)	0.0014
**Diabetic retinopathy**φ	1.2(0.6)	4.7(0.7)	0.0073

The values represent the means ± SD or percentages (SE).

The independent 2-sample t test was used for continuous variables and the χ^2^ test was used for categorical variables.

ERT, estrogen replacement therapy.

*As measured in the eye with the highest IOP value (the most affected eye); †Defined as more than 21 mmHg; ‡Defined as more than 0.5 in either the horizontal or vertical dimension; §Nuclear, cortical, posterior subcapsular, anterior polar, and mixed cataracts in individuals with the same type of opacity bilaterally; ≠Severe pterygia was defined as grade 2 and 3 pterygia; ΦDiabetic retinopathy was defined as the presence of 1 or more retinal microaneurysms or retinal blot hemorrhages with or without more severe lesions (hard exudate, soft exudate, intraretinal microvascular abnormalities, venous bleeding, neovascularization, or fibroglial proliferation).

**Table 3:** Odds ratio (95% CI) for cataract, glaucoma, pterygium, and diabetic retinopathy postmenopausal Korean women with and without ERT.

	ERT (n = 578)	Non-ERT (n = 3390)	P-value
**Cataract** §	1	1.346 (1.054,1.72)	0.0177
Cortical	1	0.966 (0.679,1.376)	0.9398
Nuclear	1	1.118 (0.871,1.435)	0.3974
Anterior polar	1	3.240 (1.123,9.351)	0.0331
Posterior subcapsular	1	1.373 (0.249,7.557)	0.7373
Mixed	1	1.504 (0.837,2.701)	0.1966
**Glaucoma**			
Increased IOP*†	1	0.477(0.095,2.384)	0.3671
Abnormal C/D ratio*‡	1	1.185(0.873,1.610)	0.2768
Disc hemorrhage	1	0.611(0.224,1.665)	0.3352
Retinal nerve fiber layer defect	1	1.703(1.044,2.777)	0.0329
**Pterygium**	1	2.202 (1.242,3.907)	0.0069
Atrophic	1	0.212 (0.072,0.626)	0.005
Flesh≠	1	3.725 (1.212,11.451)	0.0217
**Diabetic retinopathy**φ	1	4.039 (0.946,17.241)	0.0594

The values represent the multivariate-adjusted odds ratios (95% confidence interval).

Multivariate adjusted logistic regression analysis was conducted for statistical analysis. ERT, estrogen replacement therapy. *As measured in the eye with the highest value (most affected eye); † Defined as more than 21 mm Hg; ‡ Defined as more than 0.5 in either the horizontal or vertical dimension. § Nuclear, cortical, posterior subcapsular, anterior polar, and mixed cataract were recorded in individuals with the same single type of opacity present in both eyes. ≠ Flesh pterygium was defined as grade 2 and 3 pterygium, stratified according to the presence of pterygium in either eye. Φ Diabetic retinopathy was defined as the presence of 1 or more retinal microaneurysms or retinal blot hemorrhages with or without more severe lesions (hard exudates, soft exudates, intraretinal microvascular abnormalities, venous bleeding, new retinal vessels, and fibroproliferation).

#### Cataract

The prevalence of cataract was 44.2% in the ERT group and 63.3% in the non-ERT group (p<0.001). The prevalence of overall cataracts, and of nuclear and anterior polar cataracts was significantly higher in the non-ERT group. After adjusting for age, diabetes, hypertension, high cholesterol levels, and high LDL levels, the prevalence of overall cataract and anterior polar cataract remained significantly higher in the non-ERT group compared to that in the ERT group.

#### Glaucoma

Although the mean IOP was significantly higher in the ERT group (p = 0.004), it remained within the normal range. The prevalence of retinal nerve fiber layer (RNFL) defect was higher in the non-ERT group compared to that in the ERT group in both the univariate and multivariate models.

#### Pterygia

The overall pterygia prevalence was 4.5% in the ERT group and 11.9% in the non-ERT group (p<0.0001). The prevalence of atrophic pterygium was lower and that of flesh pterygium was higher in the non-ERT group compared to the ERT group in both the univariate and multivariate models.

#### Diabetic retinopathy

The prevalence of diabetic retinopathy was 1.2% and 4.7% in the ERT and non-ERT groups, respectively. The prevalence of diabetic retinopathy was significantly higher in the non-ERT group compared to that in the ERT group in the univariate model. However, after adjusting for multiple confounding factors, no differences were found in the prevalence of diabetic retinopathy in the ERT and non-ERT groups.

## Discussion

This study findings suggest a relationship between ERT and overall cataract and anterior polar cataract. Previous population-based studies have provided conflicting results regarding the effect of ERT on cataract formation. The cross-sectional data from the Beaver Dam Eye study found that postmenopausal estrogens were associated with a decreased risk of having a more severe nuclear cataract [20]. The cross-sectional data from the Blue Mountain Eye Study indicated that long-term ERT users might be protected against cortical cataracts but had an increased risk of posterior subcapsular cataracts [21]. However, long-term observational data from both studies showed little evidence of association for the exogenous estrogen exposure and lens opacities. Our cross-sectional study results differ from those of previous studies, because we found no significant differences in the prevalence of cortical, nuclear, and posterior subcapsular cataract between the 2 groups after adjusting for confounding factors. Instead, the prevalence of overall cataracts and anterior polar cataracts was significantly higher in the non-ERT group.

No relationship was found between ERT and the prevalence of high IOP, C/D ratio enlargement or disc hemorrhages. RNFL defects were less common in the ERT group (OR = 1.703). Several studies have shown lower IOP among women on ERT [22], [23], while others have reported that ERT had no effect on IOP or the risk of increased IOP [24]. One study even reported that ERT increased IOP [25]. We showed higher prevalence and larger odds ratio of RNFL defect in the non-ERT group than in the ERT group. The presence of estrogen in the vitreous may protect the optic nerve, as indicated in a previous experimental study where estrogen increased retinal blood flow in the optic nerve head region in animal models [26].

Oxidative stress leads to the development of pterygia [19], [27], and several factors, such as increasing age, male gender, and poor education have been suggested as significant risk factors for pterygium development [27], [28]. However, most of the population-based studies that investigated pterygia have included women aged 40–50 years [27]–[29], without controlling for the role of ERT. In this study, the prevalence of overall pterygia was significantly lower in the ERT group, and ERT was associated with a decreased risk of flesh pterygia. Although the pathogenesis of atrophic and flesh pterygia is not well established, fleshiness, rather than inflammation status, appears to be a significant risk factor for recurrence [29]. Estrogen in the tear film may act as a safeguard against the development of flesh pterygium by blocking oxidative stress-induced inflammation.

A previous population-based study reported that changes in the severity of retinopathy and the incidence of macular edema were unrelated to exogenous estrogen exposure [30]. Our data support these findings because no relationship was found between ERT and the prevalence of diabetic retinopathy in diabetic postmenopausal women in univariable and multivariable analyses.

This study had several limitations. First, the cross-sectional nature of the study limited our ability to perform causational analysis, and additional studies are needed to establish cause and effect between estrogen and various ocular diseases. Second, self-reported answers to questionnaires may not be entirely accurate. Thus, menopausal status was determined by using a questionnaire survey, which may be associated with information bias. Third, we included only subjects who were taking estrogen and we excluded those undergoing estrogen/progesterone treatment. However, since this study focused only on currently used medications, it is possible that some women started on one type of preparation at menopause and switched to another type several years later. In a previous study, the findings regarding the incidences of cataract or cataract surgery were similar when estrogen only and estrogen/progesterone combined treatments were analyzed separately [14]. Thus, our results may not have been affected even if a certain portion of subjects undergoing estrogen/progesterone combination treatment was included in the ERT group. Fourth, the differences between women who took estrogen versus those who did not would possibly confound the association examined in this study. The use of ERT among the subjects in this observational study is very likely related to a number of other health indicators, such as access to care, overall attitude toward health care, and willingness to accept care. Additionally, the association between ERT and systemic vascular events may result in a significant risk of confounding because physicians may choose not to prescribe ERT to those with severe vascular risk factors, and these patients are at a higher risk of diabetic retinopathy. Lastly, the duration of ERT was not surveyed in the subjects. Each 3-year increase in the duration of exogenous estrogen use was associated with a significant elevation in the risk of clinically diagnosed dry eye syndrome or severe symptoms in a population-based study [31]. If IOP elevation was due to the steroid effect of ERT in the univariable analysis of our study, the duration of ERT would be an important factor when assessing the data.

Notwithstanding the above-mentioned limitations, to our knowledge, this is the first large-scale study of its kind in an Asian population; thus, the results of this research conducted in Korea, a single-race nation, require additional investigation. The study design also utilized standardized ocular assessments performed by trained ophthalmologists, which may help to reduce the generalizability from inter-observer differences. While the limitations of this study should be considered when interpreting the evidence of a protective effect of ERT, our findings suggest that ERT protects against the development of cataract (anterior polar), pterygia (flesh type), and RNFL defects. This may be due to a protective role of estrogen in ocular tissue. The protective effect of ERT on anterior polar cataractogenesis, the development of flesh type pterygia, and RNFL defect could be explained by its known antioxidative activity [32]. The tears, aqueous humor, and vitreous may act as sources of estrogen. Although a long-term cohort study would be needed to verify the hypothesis, but these findings highlight the importance of modifiable risk factors in the prevention of ocular disease.
